# Periodicity of SNP distribution around transcription start sites

**DOI:** 10.1186/1471-2164-7-66

**Published:** 2006-04-03

**Authors:** Koichiro Higasa, Kenshi Hayashi

**Affiliations:** 1 Division of Genome Analysis, Research Center for Genetic Information, Medical Institute of Bioregulation, Kyushu University, Maidashi 3-1-1, Higashi-ku, Fukuoka 812-8582 Fukuoka, Japan

## Abstract

**Background:**

Several millions single nucleotide polymorphisms (SNPs) have already been collected and deposited in public databases and these are important resources not only for use as markers to identify disease-associated genes, but also to understand the mechanisms that underlie the genome diversification.

**Results:**

A spectrum analysis of SNP density distribution in the genomic regions around transcription start sites (TSSs) revealed a remarkable periodicity of 146 nucleotides. This periodicity was observed in the regions that were associated with CpG islands (CGIs), but not in the regions without CpG islands (nonCGIs). An analysis of the sequence divergence of the same genomic regions between humans and chimpanzees also revealed a similar periodical pattern in CGI. The occurrences of any mono- or di-nucleotide sequences in these regions did not reveal such a periodicity, thus indicating that an interpretation of this periodicity solely based on the sequence-dependent susceptibility to mutation is highly unlikely.

**Conclusion:**

The periodical patterns of nucleotide variability suggest the location of nucleosomes that are phased at TSS, and can be viewed as the genetic footprint of the chromatin state that has been maintained throughout mammalian evolutionary history. The results suggest the possible involvement of the nucleosome structure in the promoter function, and also a fundamental functional/structural difference between the two promoter classes, i.e., those with and without CGIs.

## Background

Several million single nucleotide polymorphisms (SNPs) have already been collected and deposited in public databases [1] and these are important resources not only for use as markers to identify disease-associated genes [2], but also for an understanding of the mechanisms that underlie the diversification of the organism. The nucleotide diversity of human genome sequence appears to fluctuate from region to region [3-5]. The majority of the SNPs are believed to have no biological consequence, and therefore their diversity is primarily determined by the mutation rate within the germ cells, although it may be affected by the selective pressure that operates at the individual level [6]. In this study, we used a spectral analysis approach to identify the pattern of nucleotide variability around the transcription start sites (TSSs), and survey its biological implication.

## Results

### Nucleotide diversity

**Figure 1:**
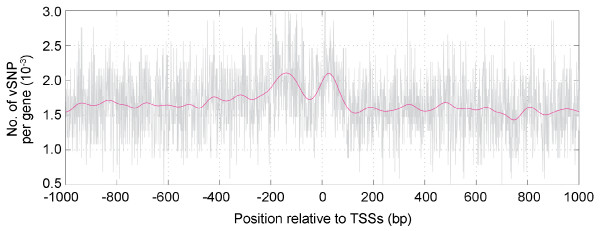
**Distribution of SNPs around TSSs.**The distribution of the density of validated SNPs (no. of vSNPs per gene) at the positions relative to the TSSs of 10,171 genes are shown (gray). Noise filtering was performed using FFT. After the SNP density data was transformed to the frequency domain by means of an FFT, the one-sided low-pass Hanning filter for components below 50 nucleotides was applied. The denoised curve was obtained by the inverse FFT of the filtered array (magenta).

We first noticed a periodicity of the nucleotide diversity around TSSs using the genotype data obtained from the dbQSNP database (version 11) [7], in which approximately 10^4 ^SNPs located around the 1.2 kb promoter regions of 4 × 10^3 ^genes have been identified and mapped on the Reference Human Genome Sequence [8]. These SNPs were discovered by re-sequencing the DNA of eight individuals. In this database, all data including the regions without detectable SNPs have been described. Thus, the per-nucleotide diversity (π) of each nucleotide position relative to TSS can be estimated by aligning each of the examined sequences at TSS, since the examined number of individuals are known [6]. A striking feature of the distribution of π was its waviness (data not shown). We expanded the analysis using the TSS regions described in DBTSS, in which approximately 1.3 × 10^4 ^TSSs have been identified by mapping the 5'-end sequences of more than 4 × 10^5 ^full-length cDNA clones onto the genome [9,10]. We further selected 10,171 sites, which were the most frequently used TSSs for each of the genes (the genomic site with the largest number of the 5' ends for each gene defined in DBTSS), to avoid overrepresentation of the genes with multiple promoters. TSS regions, i.e., the sequences 3 kb in both directions from the start sites, were collected from the reference human genome sequence, and 97,041 validated SNPs (defined in dbSNP) that fell in these regions were mapped (approximately 1 SNP per 600 nucleotides). Next, the SNPs at each nucleotide position (relative to TSS) were counted to obtain the distribution of the SNP density around the TSS (Fig. 1). In this case, the per-nucleotide diversity could not be estimated, because the number of chromosomes examined to find the SNPs is unknown. However, the SNP density can be regarded as an indicator of the nucleotide diversity, since an ascertainment bias is unlikely to affect the local distribution of SNPs at this resolution. The wavy nature of the distribution similar to the per-nucleotide diversity described above was also observed.

### Spectrum analysis

**Figure 2:**
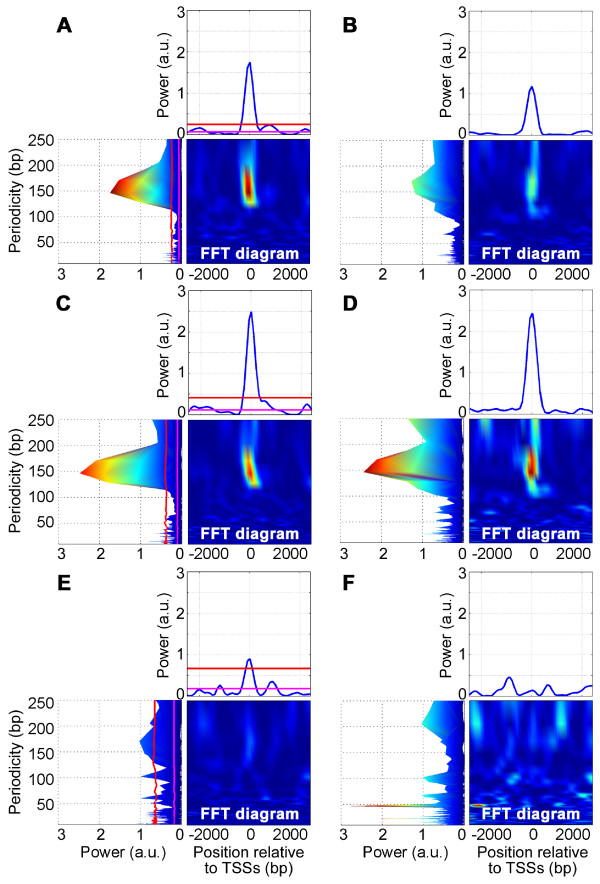
**Spectrum analysis by Fast Fourier transformation**. Spectra of distributions of SNP density (A, C, and E) and nucleotide divergence between humans and chimpanzees (B, D, and F) of three TSS categories; all TSS (A and B), CGI-TSSs (C and D) and nonCGI-TSSs (E and F). The side view and sectional view at the periodicity 146 nucleotides of the FFT diagrams are shown on the left and top of the diagram panels, respectively. The magenta and red lines are the means and the 99 % confidence intervals of the power values. The number of sequences analyzed are 10,171 (A), 6,329 (C), and 3,842 (E). The diagrams and their side views of SNP density (A, C and E) are dynamically colored according to the Z-scores, while those of divergence (B, D and F) are colored according to the power in arbitrary units, which are the square of coefficients for the polynomials of the trigonometric functions in the FFT. The color range for SNP density goes from blue to red, corresponding to 0 to 25 in Z-score. Those for divergence correspond to 0 to 3, respectively, in power value (a.u.).

A spectrum analysis by Fast Fourier transformation (FFT) of the SNP density distribution revealed a remarkable periodicity around the TSSs, with the most conspicuous peak of power value 1.7 × 10^-3 ^at the wave length 146 nucleotides, and this periodicity persisted at positions ranging approximately between -200 and +200 (Fig. 2A). To determine the statistical significance of this periodicity, we estimated the mean and standard deviation of the power values for each wavelength in random spectra of an equivalent number of data set. Namely, we carried out 10^3 ^simulations, each consisting of the distribution of validated SNPs around randomly chosen 10,171 genomic sites. As shown in Figure 2A, the power of the peak at the 146 nucleotide was statistically significant, since the power value fell far outside the three times the standard deviation of the power for the random sites.

In many genes, the position of their TSSs fluctuates more or less [11], and the degree of this fluctuation could cancel out the effects of phasing to TSSs. We thus evaluated the periodicity in two classes of genes; those with small fluctuations and those with large fluctuations. We defined the extent of fluctuation as follows. We chose 4,660 genes for which more than 10 oligo-capped clones were mapped. They were then divided into two halves according to the start site fluctuation, which were estimated by the value of the standard deviation of the start positions. A spectrum analysis of the SNP density distribution of these two classes revealed a stronger signal (4.5 × 10^-3^) at the 146 bp periodicity for the TSSs with small fluctuations than for those with large fluctuations (1.5 × 10^-3^).

This periodical distribution of SNP density raised the question of whether they might be caused by the sequence features around TSSs. We examined the distribution of each of the four nucleotides, or all 16 dinucleotide that include CpG dinucleotide that is known to have a higher mutation rate (see Additional file 1). We found various periodical distributions both for some mono- and di-nucleotide sequences, but none of them showed the 146 nucleotides periodicity. The sequence-dependent susceptibility to mutation is thus not considered to explain the periodicity of the diversity profile.

### CpG island and periodicity

In the mammalian genome, CpG dinucleotide is suppressed (depleted), because most of the C in CpG are methylated at the C5 position by the CpG methylase activity, that in turn tends to be mutated to T by spontaneous deamination. The CpG islands (CGIs) are exceptions, where their local C/G contents are high, and the dinucleotides are not depleted [12]. The island is frequently located in the vicinity of TSS [13].

**Figure 3:**
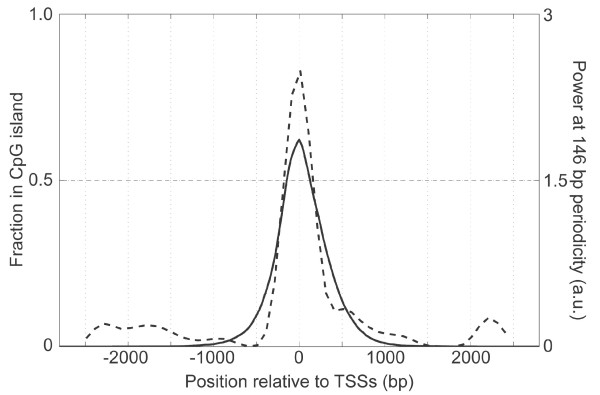
**Co-localization of CpG island and the 146 nucleotides periodicity**. Occupancy of CpG islands (solid line, scale on the left) and the power of the 146 nucleotides periodicity of SNP density (dashed line, scale on the right) around the TSSs are shown. a.u., power in arbitrary units.

We mapped CGIs within the 6 kb TSS regions by NewCpGreport program (EMBOSS v.2.10.0 package) [14,15] using default parameters, i.e., the C/G content greater than 50 %, the observed/expected ratio of the CpG appearance greater than 0.6 and the size of the island longer than 200 nucleotides. The regions were then classified into two groups, the TSSs within CGI (CGI-TSSs) or not (nonCGI-TSSs). Among the 10,171 TSSs, 65 % were CGI-TSSs (Fig. 3), which closely agreed with the previously reported values [16]. A spectrum analysis of the SNP density distribution of the two classes of regions revealed a stronger signal (2.5 × 10^-3^) at the 146 nucleotides periodicity for the CGI-TSSs, but none for the nonCGI-TSSs (Fig. 2C and 2E). We also noticed that the range of the genome around TSS covered by CGI roughly matched the range where the 146 nucleotides periodicity of SNP density is observed (Fig. 3).

### Periodicity of human-chimpanzee divergence

The genome diversity observable within one species (i.e., human) is limited because of various factors e.g., short time (2 to 3 × 10^4 ^years ago) since the establishment of Homo sapiens, the population bottle-neck, for assessing the variability of the genome sequence. On the other hand, the genome divergence between closely related species, i.e., human and chimpanzee, can yield more information about the genome variability, because the mutations are accumulated and fixed within each population since the separation of the two species 5 million years ago, and yet, they are close enough so that the genome sequences can be reliably aligned to precisely determine the locations of the changed nucleotides. We identified the TSS regions of the chimpanzee genome by BLAT [17] searching using the human TSSs as query sequences. Approximately 90% (9,087) of the 10,171 human TSS regions could be aligned with confidence, and 61% (5,529) of them were with CGI. A total of 400,285 divergent sites could be mapped in these regions. The spectrum analysis of nucleotide divergence between humans and chimpanzees again showed the 146 nucleotides periodicity, which was derived solely from the CGI-TSS regions (Fig. 2B, 2D and 2F).

## Discussion

We have shown that both the SNP density distribution and the nucleotide divergence profile between species (human and chimpanzee) are periodical around the TSSs, with its wave length of 146 nucleotides, which is identical to the length of DNA that wraps around the nucleosome. This periodicity comes solely from the TSS regions with CGI, and the range the periodicity is observed is roughly the same as the CGIs occupy, i.e., 0.4 kb or 2 to 3 nucleosome units. We are thus tempted to propose, that the CGIs are sites where nucleosomes are tightly packed and phased to the TSSs, and that the nucleotide variability is positionally biased within the nucleosomal structure. Several previous reports have also supported this idea, i.e., nucleosome positioning in the promoter region has been experimentally explored for a few human genes [18-21]. Among these promoters, those with CGI were shown to be organized into a phased array of nucleosomes [18,19], while those without CGI carried only a single nucleosome located at some distances from their TSSs [20,21].

One possible explanation for the intra-nucleosomal bias of nucleotide variability is the local difference of mutation rate in germ cells. The nucleosome structure can locally affect the mutation rate, because its determinants, e.g., mutagen accessibility, depurination rate, or the efficiency of damage recognition and repair may be position-dependent. While assuming that the periodicity of nucleotide variability is ascribed to that of the mutation rate, it follows that the CGI-TSSs are in a nucleosome-packed and phased state, but nonCGI-TSSs are not, in the germ cell lineage.

The CGIs have originally been recognized to be the characteristic promoter regions of housekeeping genes, whose expression is necessary for the maintenance of cell physiology, and so, are widely expressed regardless of the cell types. However, the distinction between housekeeping genes vs. non-housekeeping (or accessory) genes has been somewhat arbitrary. The germ line cells are, by definition, in an undifferentiated state (as far as gene expression pattern is concerned), and so, the genes with CGI-TSSs are likely to be expressed. It is thus a plausible idea that CGIs are involved in the expression of genes essential for the functioning and survival of germ cells, and that an ordered nucleosome location is required for this expression.

An alternative explanation for the bias of the nucleotide variability within the nucleosome structure is the sequence constraint that acts at the individual level. The recognition sequences of various transcription factors (and so, sites of conservative pressure) may thus be located at a particular site (or side) of the nucleosomal structure. According to this scenario, some additional assumptions are needed to explain the absence of periodicity of the nucleotide variability in the nonCGI-TSSs.

The sequence features that are preferred for winding around the nucleosome have been previously amply described. Using experimentally derived nucleosomal DNA sequences, some dinucleotides, e.g., AA or TT, have been shown to appear at distances that are multiples of 10.1 to 10.5 nucleotides, which are the pitches of the helix of double-stranded DNA wrapping around the core histones [22-25]. Using the dataset of the TSS regions described here, we examined the periodicity of the distances by an autocorrelation function [24] for the two dinucleotides mentioned above. We found significant periodicities of the ten-nucleotide for TT distances in CGI-TSSs but not in nonCGI-TSSs (see Additional file 2). These were located in the region where the periodical nucleotide variability was observed (Fig. 3). These results further support the idea that CGI-TSSs are likely to be organized into a phased array of nucleosomes but not nonCGI-TSSs. In addition, these nucleosome positionings were consistent with those identified by others as the sites of micrococcal nuclease resistance for the gene with CGI-TSS [18]. The distribution of the nucleosomal DNA signal detected in this study may also suggest the general chromatin architecture around CGI-TSSs.

Ioshikhes et al., have shown that certain transcription factor binding sites may also show the same distance periodicity (10.1 – 10.5 nucleotides) [26]. We could not detect statistically significant periodicity of SNP density of the 10 nucleotides around TSSs (see Additional file 3).

We showed here, that nucleotide variability can be viewed as the genetic footprint of the chromatin state around TSSs. The direct proof of the two possible explanations here may be provided by showing the phased and packed localization of nucleosomes around the TSSs in germ cells. This requires the development of a method that enables an examination of the spatial relationship of histone molecules with specific genomic sites at the resolution of the nucleotide level, and also at the sensitivity of the detection of the events occurring in single to a few cell level, since the germ cells are few in number.

## Conclusion

We herein reported a periodical pattern of SNP density distribution around the transcription start sites (TSSs) that are associated with CpG islands (CGIs). The wavelength of the periodicity matches the length of DNA in the nucleosome. The sequence divergence of the same genomic region between humans and chimpanzees also revealed a similar periodical pattern. These results indicated that nucleotide variability can be viewed as the genetic footprint of the chromatin state around the TSSs, which has remained throughout mammalian evolutionary history.

## Methods

### Definition of Transcription Start Sites (TSSs)

The human promoter sequences were downloaded from the DataBase of Transcriptional Start Sites (DBTSS version 4) [27], which contained information of the exact genomic positions (on the Reference Human Genome Sequence, Build 34) of the TSSs and the adjacent regions for 12,763 human genes. We re-mapped the TSSs onto the reference sequence of the latest version (i.e., Build 35) [28], and the alternative TSSs were excluded, so that a total of 10,171 TSSs were subjected to the analysis. The 6,001 nucleotides sequences between -3,000 nucleotides and +3,000 nucleotides to TSSs were obtained from the reference sequence. The accession numbers of the reference sequences and sequences around TSSs are available in Additional files 4, 5 and 6, respectively.

### Polymorphism data

The chromosomal positions of validated SNPs around the TSSs were obtained from dbSNP (Build 124) [29], and then were converted to those relative to TSS. The occurrences of validated SNPs at each nucleotide position were summed up to obtain the SNP density. These data are available in an Additional file 7.

### Spectrum analysis

The position-dependent frequency content and power spectra were calculated by the windowed Fast Fourier Transform using the MATLAB® (The MathWorks, Inc., USA). Sliding windows (1,024 nucleotides) at a step of 100 nucleotides were adopted to provide an appropriate balance between the spatial scope and the resolution of frequency for the analysis. The Hanning function was applied to each window to avoid an artifact of discontinuity at the ends. The Matlab scripts and input files for each category of TSS's (i.e., all TSS, CGI-TSSs, and nonCGI-TSSs) are available as Additional file 8.

### Statistical evaluation of periodicity

The power spectra were evaluated by the occurrence of the periodicity in 10^3 ^random data sets. Each set was composed of N random sequences of length 6,001 nucleotides picked from random positions in the reference sequence (Build 35), where N is the number of TSS regions in each category. The mean and standard deviation of the power was obtained for each periodicity. The confidence interval of 99% was assumed to be 2.58 times the standard deviation (Z-score = 2.58) from the mean, in the distributions of the power values.

### Sequence alignment of TSSs between human and chimpanzee

TSS regions of the chimpanzee genome were obtained by BLAT search (version 32) [30] of the genome [31] using the 10,171 human TSS regions as query. Alignments with the score below 400 were excluded. Most of the human TSSs (10,099/10,171) could be aligned with the chimpanzee genome. To eliminate the alignment errors in the process of determination of mismatch positions in sequence pairs with insertions/deletions, the alignment was limited between the TSSs and most proximal gaps of more than two nucleotides. Consequently, the number of aligned sequences varied depending on the positions, from 9,087 at TSS to 2,738 and 3,243 at -3,000 and +3,000, respectively. The mismatch frequencies at each nucleotide position were assumed to be the nucleotide divergence between the human and chimpanzee TSS regions.

## Authors' contributions

KHI conceived the study and carried out the analysis. KHA participated in its design and coordination, and helped to draft the manuscript.

## Supplementary Material

Additional File 1 **Periodicity of mono- and di-nucleotide sequences around TSSs**. The spectra of nucleotide frequency for three TSS categories; all TSS (A), CGI-TSSs (B) and nonCGI-TSSs (C). The side views are shown on the left of the diagram panels. The magenta and red lines are the means and 99 % confidence intervals of the power values that were determined from the distributions of the values in simulations using randomly chosen genomic positions as described in the text. The dynamic color range goes from blue to red, corresponding to 0 and 200 in the Z-score, respectively. a.u., arbitrary units. Click here for file

Additional File 2 **Autocorrelation function maps for AA and TT dinucleotides around the TSSs**. Autocorrelation function maps around CGI-TSSs (A and B) and nonCGI-TSSs (C and D). After the calculation of autocorrelation function in the sliding windows (146 nucleotides) with a step of 5 nucleotides from -3,000 to +3,000 nucleotides relative to the TSSs, the moving average over 3 distances for each function was applied to remove a period 3 due to the coding region. The position of the window's center is represented. The statistical significance of functions in the each window was evaluated by mean and standard deviation calculated from those of all sliding windows. The dynamic color range goes from blue to red, corresponding to 0 and 10 in the negative log of probability p, respectively. The contour interval is 1.0. The zones of possible nucleosome occupancy as judged from the 10 nucleotide periodicity are in pink. Click here for file

Additional File 3 **Spectrum analysis of the SNP density in the short wave-length range**. The spectra of SNP density distribution for three TSS categories; all TSS (A), CGI-TSSs (B) and nonCGI-TSSs (C). Short sliding windows (128 nucleotides) at a step of 5 nucleotides from -3,000 to +3,000 relative to TSSs were adopted. The side views are shown on the left of the diagram panels. The magenta and red lines are the means and 99 % confidence intervals of the power values that were determined from the distributions of the values in simulations using randomly chosen genomic positions as described in the text. The dynamic color range goes from blue to red, corresponding to 0 and 25 in the Z-score, respectively. a.u., arbitrary units. Click here for file

Additional File 4 **List of NM ID in each category**. List of the accession numbers of mRNA Reference Sequences (NM ID) in each TSS category. Click here for file

Additional File 5 **Sequence around TSSs (Part 1)**. Fasta formatted file containing the sequence from position -3,000 to +3,000 relative to TSS. Click here for file

Additional File 6 **Sequence around TSSs (Parts 2)**. Fasta formatted file containing the sequence from position -3,000 to +3,000 relative to TSS. Click here for file

Additional File 7 **Accession numbers of the Reference Sequences and SNP information**. List of the accession numbers of the 10,171 unique human mRNA Reference Sequences (NM ID) used to analyze the distribution of the SNP density in this study; includes the associated chromosome number, contig ID, position in contig, and SNP information from position -3,000 to +3,000 relative to TSSs (1 and 0 indicate hit and no-hit of validated SNP at each position, respectively) Click here for file

Additional File 8 **Matlab script and input files**. The input files for Matlab script is a 2-column 6001-line table in ASCII format, in which the first and second columns represent the relative position to TSSs and the number of SNPs at each position, respectively. For nucleotide divergence, an additional third column represents the number of aligned sequences between the humans and chimpanzees at each position relative to TSSs. Click here for file
