# Supplementary figures and images for: Periodicity of SNP distribution around transcription start sites
Source: BMC Genomics. 2006 Apr 3;7:66. doi: 10.1186/1471-2164-7-66 (PMC1448210; doi:10.1186/1471-2164-7-66)

**A**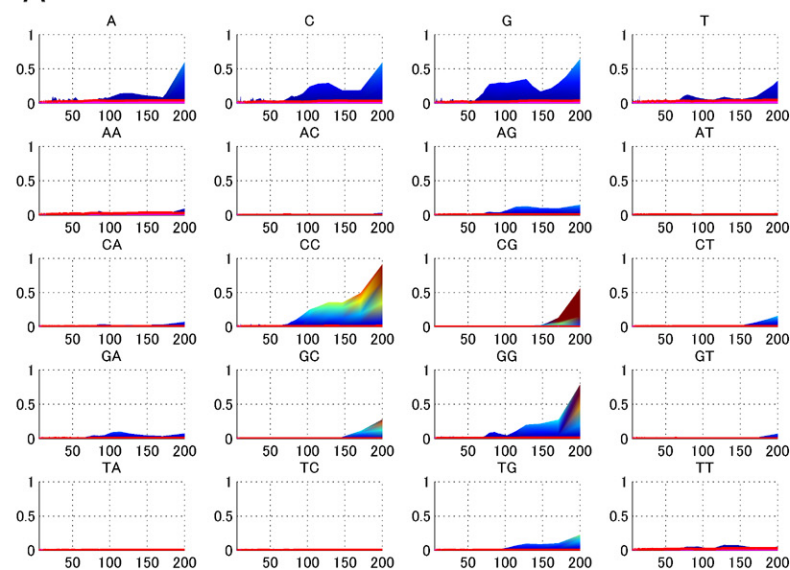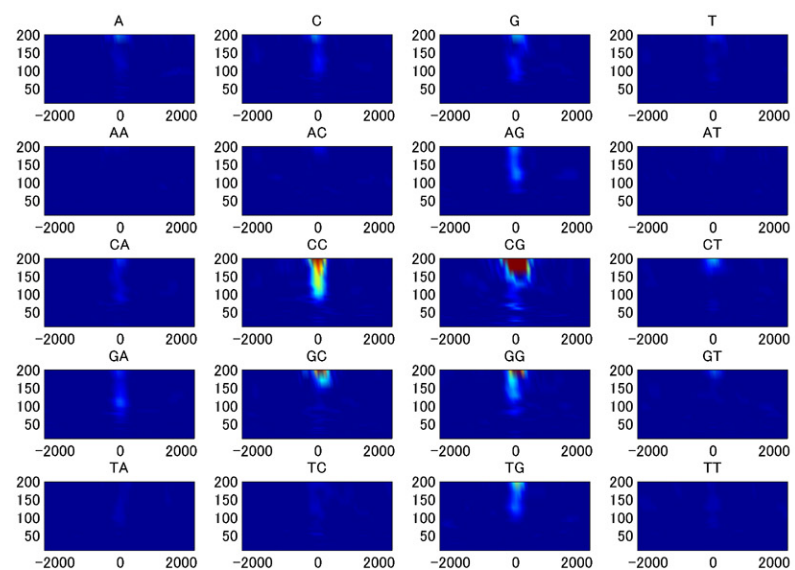**B**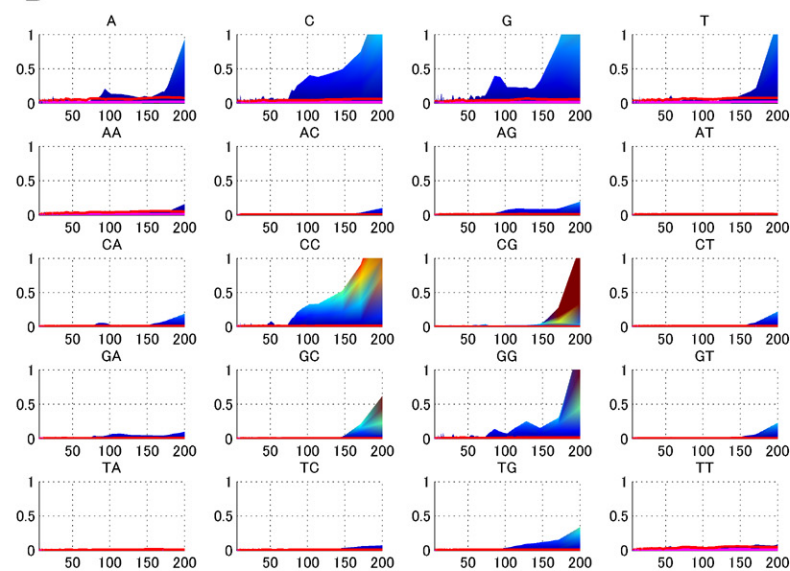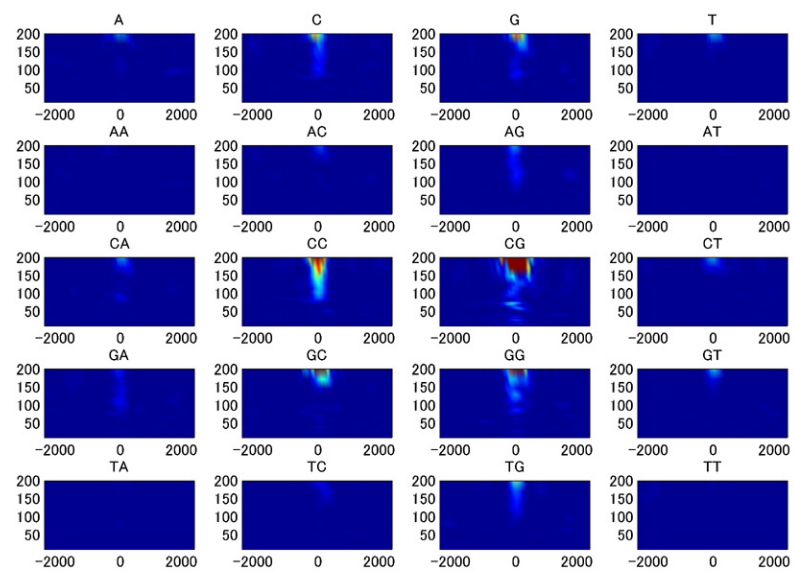**C**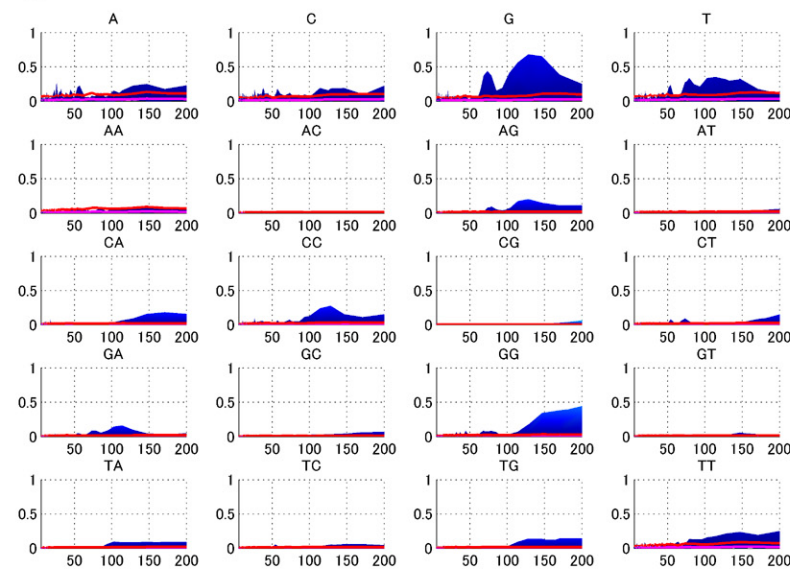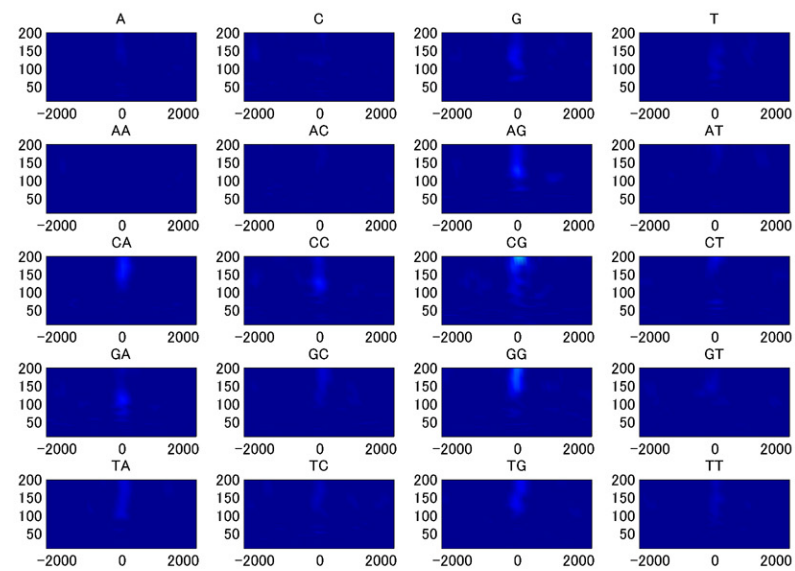

Supplement: Additional File 1 — Periodicity of mono- and di-nucleotide sequences around TSSs. The spectra of nucleotide frequency for three TSS categories; all TSS (A), CGI-TSSs (B) and nonCGI-TSSs (C). The side views are shown on the left of the diagram panels. The magenta and red lines are the means and 99 % confidence intervals of the power values that were determined from the distributions of the values in simulations using randomly chosen genomic positions as described in the text. The dynamic color range goes from blue to red, corresponding to 0 and 200 in the Z-score, respectively. a.u., arbitrary units. [file 1471-2164-7-66-S1.pdf]

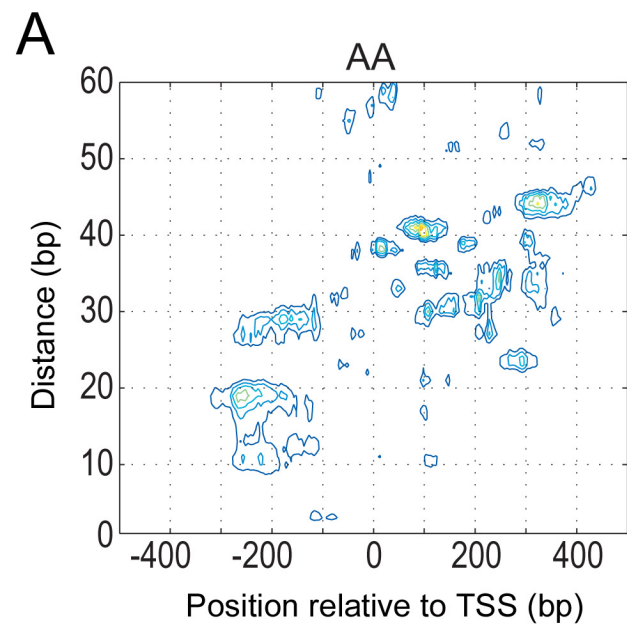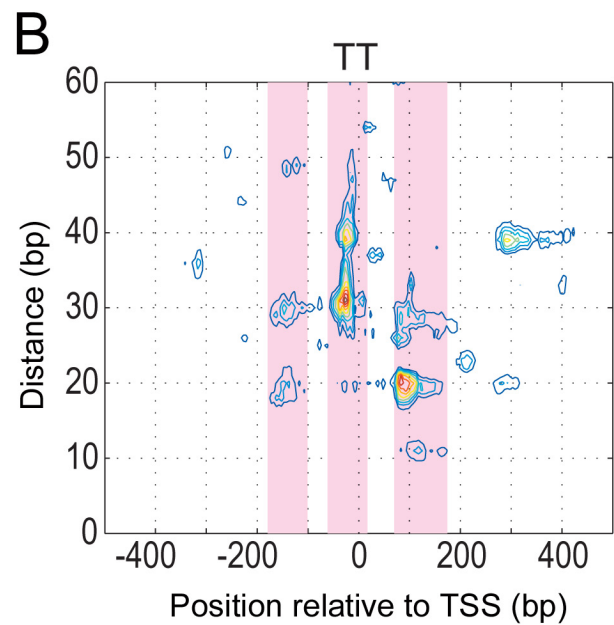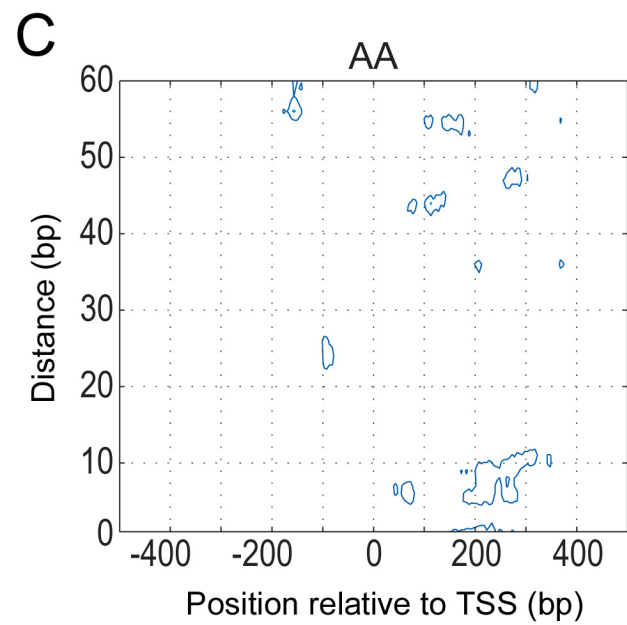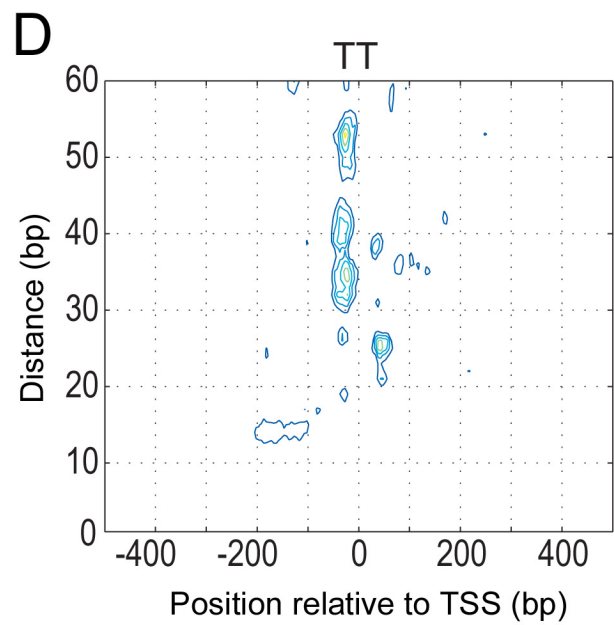

Supplement: Additional File 2 — Autocorrelation function maps for AA and TT dinucleotides around the TSSs. Autocorrelation function maps around CGI-TSSs (A and B) and nonCGI-TSSs (C and D). After the calculation of autocorrelation function in the sliding windows (146 nucleotides) with a step of 5 nucleotides from -3,000 to +3,000 nucleotides relative to the TSSs, the moving average over 3 distances for each function was applied to remove a period 3 due to the coding region. The position of the window's center is represented. The statistical significance of functions in the each window was evaluated by mean and standard deviation calculated from those of all sliding windows. The dynamic color range goes from blue to red, corresponding to 0 and 10 in the negative log of probability p, respectively. The contour interval is 1.0. The zones of possible nucleosome occupancy as judged from the 10 nucleotide periodicity are in pink. [file 1471-2164-7-66-S2.pdf]

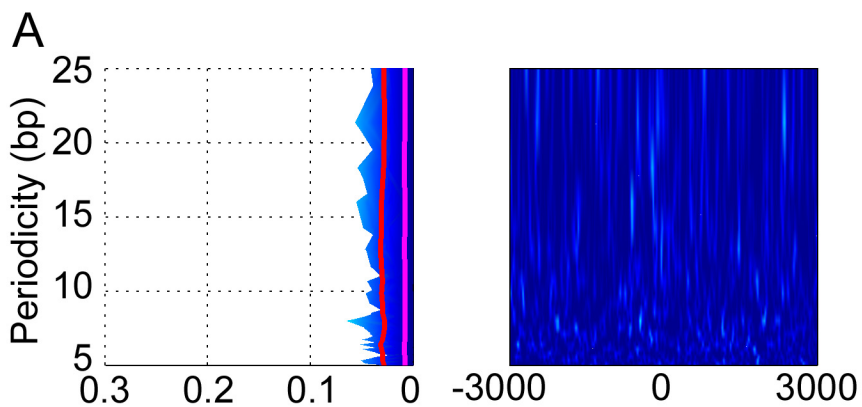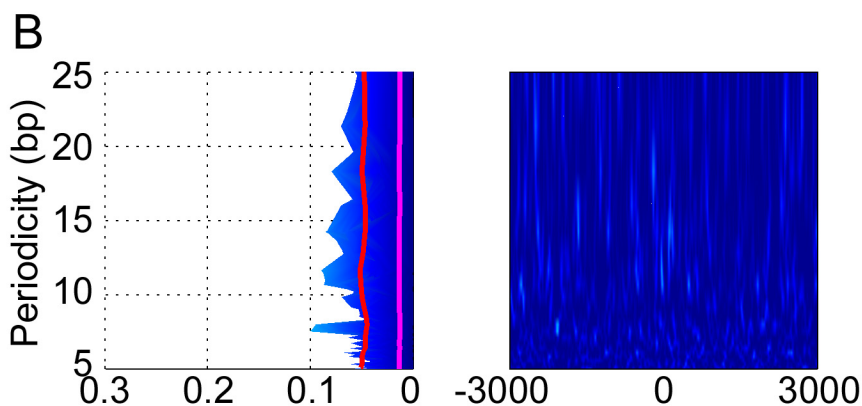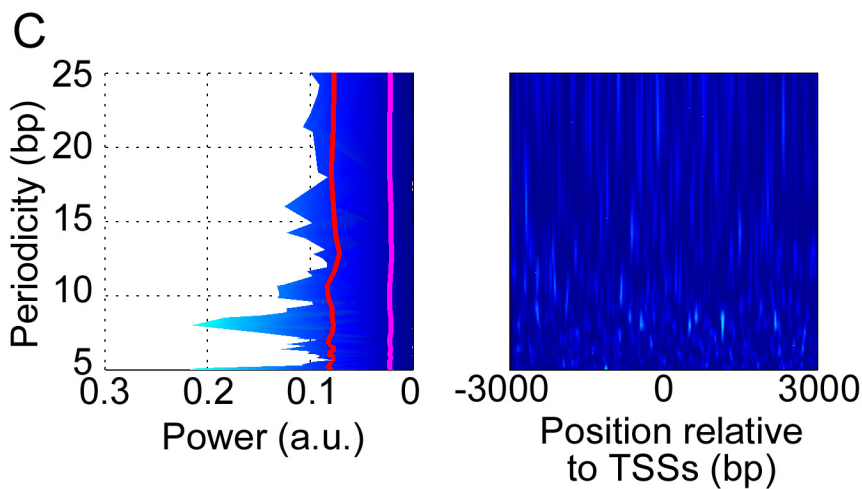

Supplement: Additional File 3 — Spectrum analysis of the SNP density in the short wave-length range. The spectra of SNP density distribution for three TSS categories; all TSS (A), CGI-TSSs (B) and nonCGI-TSSs (C). Short sliding windows (128 nucleotides) at a step of 5 nucleotides from -3,000 to +3,000 relative to TSSs were adopted. The side views are shown on the left of the diagram panels. The magenta and red lines are the means and 99 % confidence intervals of the power values that were determined from the distributions of the values in simulations using randomly chosen genomic positions as described in the text. The dynamic color range goes from blue to red, corresponding to 0 and 25 in the Z-score, respectively. a.u., arbitrary units. [file 1471-2164-7-66-S3.pdf]
